# A Prospective Study on the Clinical Significance of Infections in a Hospital Setting Among the Cirrhotic Patients and Their Outcomes

**DOI:** 10.7759/cureus.37912

**Published:** 2023-04-21

**Authors:** Swarup K Patnaik, Sambedana Mohanty, Debakanta Mishra, Manjit Kanungo, Srinith Patil, Ram Gopal Teja, Kanishka Uthansingh, Jimmy Narayan, Manoj K Sahu, Girish K Pati

**Affiliations:** 1 Gastroenterology, Siksha 'O' Anusandhan Deemed to be University Institute of Medical Sciences and SUM Hospital, Bhubaneswar, IND; 2 Community Medicine, Siksha 'O' Anusandhan Deemed to be University Institute of Medical Sciences and SUM Hospital, Bhubaneswar, IND; 3 Gastroenterology, ESIC Medical College, Gulbarga, IND; 4 Gastroenterology, Delta Hospitals, Rajahmundry, IND

**Keywords:** acute-on-chronic liver failure, nosocomial infection, health care associated infection, community acquired infection, alcoholic liver disease

## Abstract

Aim and objectives

The infection of microbial agents in cirrhosis has increased due to poor immunity, which increases morbidities and mortalities worldwide. The present study aimed to assess the incidence, the type of infections, the pattern of resistance, and the course of hospitalization among cirrhotic patients in the Eastern coastal region.

Methodology

The study was a descriptive cross-sectional study, and the current study was undertaken for 24 months at the Department of Gastroenterology and Hepatobiliary Sciences, IMS, and SUM. Hospital, Bhubaneswar. Consecutive cirrhotic patients admitted with bacterial infection were prospectively evaluated, and the infection patterns were accessed. The data were collected in a well-structured proforma designed by our study team.

Results

Out of the total 200 cases, a fraction of 72.5% of males outnumbered the females; the mean age of presentation was 59 ± 12 years. A fraction of 59% of cases had the habit of consuming alcohol which amounted to the predominant etiological factor for cirrhosis, followed by non-alcoholic steatohepatitis (NASH). Urinary tract infection (UTI) and spontaneous bacterial peritonitis (SBP) were more common types of infections in the healthcare-associated (HCA) group; however, pneumonia and skin and soft tissue infections (SSTI) were predominant types of infections in community-acquired (CA) group. The model for end-stage liver disease (MELD) scores were not significantly different amongst the three groups with infections at the time of Diagnosis infection and at the time of hospitalization. However, the MELD scores were substantially higher at the time of infection diagnosis than the MELD scores at the time of admission amongst the three groups with infection.

Conclusion

The present study showed that infections in cirrhosis were relatively common. Due to increasing resistance patterns, the judicious usage of antibiotics in cirrhosis could be the need of the hour.

## Introduction

Patients with liver cirrhosis have an altered immune system, predisposing them to various infections. Cirrhosis-associated immune dysfunction syndrome (CAIDS) results from the overwhelming activation of proinflammatory cytokines in cirrhosis and aberrant portosystemic shunting, which lead to inappropriate and ineffective bacterial clearance [1]. Cirrhosis patients with bacterial infections have a four-fold increase in mortality compared to those in the non-cirrhotic population [2]. Delayed intestinal transit time, bacterial overgrowth, increases in proinflammatory cytokines and nitric oxide, and portosystemic shunting contributes to increased bacterial translocation to mesenteric lymph nodes, systemic circulation through leaky gut syndrome, and ultimately the development of ascites and cirrhosis [3]. The reticuloendothelial (RE) cell dysfunction in cirrhosis leads to aberrant and inappropriate monocyte and neutrophil activation and trafficking and poor bacterial phagocytosis. Insufficient phagocytic and bactericidal activity and inefficient opsonization activity occur due to decreased albumin levels in cirrhosis, which is further associated with lower blood levels of immunoglobulins and complement factors, compromising immunity further and increased risk for bacterial infection [4]. The immunocompromised state in cirrhosis is further complicated by associated malnutrition due to poor appetite, immunosuppressive medications, and alcohol consumption, which lead to poor activities of T, B, and natural killer cells [5].

Bacterial infection is relatively common in cirrhosis and increases the morbidity and mortality of the disease [6]. Cirrhosis patients are often immunocompromised or develop hypo-albuminemia, and they are more prone to spontaneous bacterial peritonitis (SBP), hospital-acquired infections, and infections by un-common pathogens. Once infection sets in, serious complications such as shock, acute-on-chronic liver failure (ACLF), renal failure, and death may result because of the altered and inappropriate response of proinflammatory cytokines to infection, especially in cases of pre-existing hemodynamic dysfunction. Unfortunately, infections due to resistant bacteria have increased significantly in healthcare-associated (HA) settings. In addition to precipitating complications, hepatic encephalopathy (HE) and bacterial infections are often responsible for developing acute kidney injury (AKI) and ACLF, which can delist patients seeking a liver transplant and increase mortality. One of the major concerns in hospitalized cirrhosis patients is the development of nosocomial infections [7]. Although Gram-positive bacterial infections are not reasonably common in hospitalized cirrhosis patients, many patients suffer from infections caused by Gram-negative bacteria under the Enterobacteriaceae family, as reported in a prior study [8]. One-third of patients hospitalized with cirrhosis develop at least one infection, with two-thirds of such incidents resulting from healthcare-associated or nosocomial conditions [9-10].

SBP accounts for most infections in cirrhosis patients at about 25%-31%, followed by urinary tract infection (UTI) in 20-25% of patients and pneumonia in 15-21% of cases [11]. As there are limited data on infections and their impact on clinical outcomes in Indian cirrhosis patients, the current study aimed to determine the prevalence and modes of bacterial infections in cirrhosis patients in an Indian hospital and to investigate trends in hospitalization duration and prognosis.

## Materials and methods

Patients and study design

Approval was obtained from the Institutional Ethical Committee before the commencement of the study (Ref. No. DRI/IMS.SH/SOA/2021/029). Written informed consent was obtained from each participant before initiating the study. The current study was a cross-sectional study conducted from November 2019 to November 2021 at the Department of Gastroenterology and Hepatobiliary Sciences, IMS & SUM Hospital, Bhubaneswar, India. The study included all the cirrhosis patients admitted to the department during the abovementioned period.

Operational definitions

Data were collected on the modes of infection among the cirrhosis patients, which were defined as follows: (1) Community-acquired (CA) infection, which was diagnosed within 48 hours of hospitalization with no prior hospitalization within the last six months; (2) Healthcare-associated (HCA) infection, which was diagnosed during hospitalization or within 48 hours of hospitalization with prior exposure to health-care environments; (3) Nosocomial (NC) infection, which was diagnosed after 48 hours of hospitalization.

To predict survival, MELD looks at the blood bilirubin, serum creatinine, and INR levels of the patient were calculated. The formula used to calculate it is as follows: MELD scores are presented as whole numbers. The sepsis and no sepsis were diagnosed by identification of the pathogens through urine and stool culture.

Inclusion criteria

The final study protocol included cirrhosis patients aged 18 years or older with confirmed bacterial infections. The subjects were evaluated at baseline and searched for possible infections at admission and during hospitalization Patients who developed an infection before hospitalization were included in the study, as were those who developed an infection after hospitalization. Only patients who consented to participate in the study were included.

Exclusion criteria

Patients with HIV, immunocompromised states, pregnant women, and patients younger than 18 years were excluded from the study.

Data collection

The data were collected primarily from the patients to calculate the MELD scores and CRFs from all the eligible patients using the collected data.

Data analysis

The data were analyzed by SPSS version 26 software; continuous data were expressed as a mean ± SD (standard deviation), and categorical data were expressed as proportions and percentages. Differences among continuous data between groups were analyzed by Student's t-test or the Mann-Whitney U test, whereas Fisher's exact test analyzed differences among categorical data. One-way or two-way ANOVA was used for analyses within groups as required. In the analyses, p<0.05 was considered significant.

## Results

**Figure 1:**
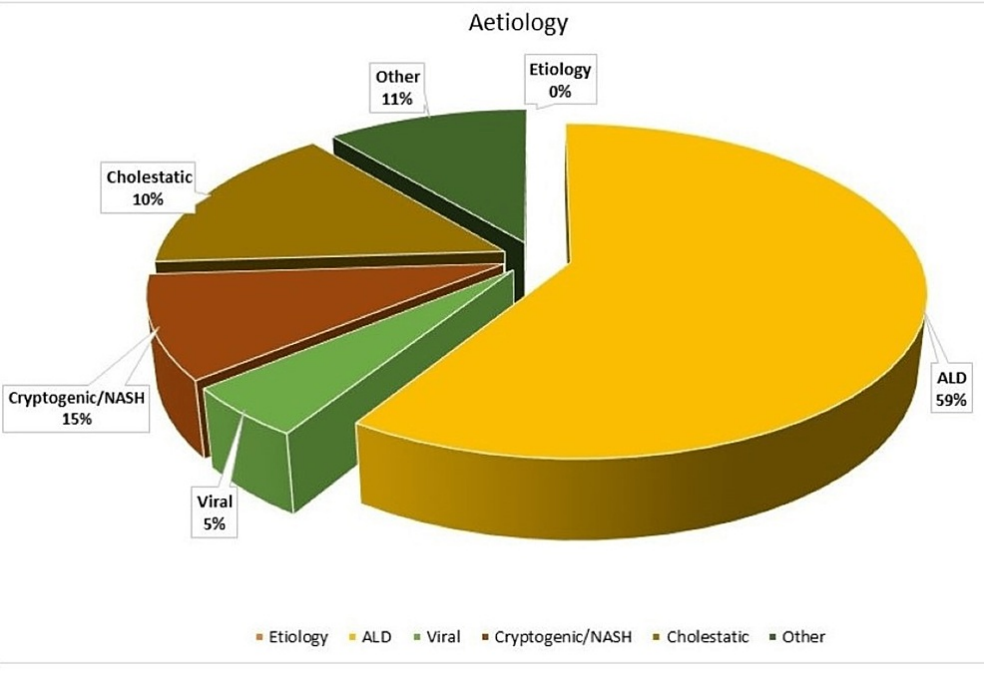
Aetiological pattern of cirrhosis ALD: alcoholic liver disease; NASH: non-alcoholic steatohepatitis.

We obtained data from 200 cases, of which there was a higher proportion of male patients (72.5%), and the mean age at presentation was 59 ± 12 years. Figure 1 provides an illustration of the etiological patterns of cirrhosis in patients.

In our study population, 59% of patients had a history of significant habits of alcohol, which was the predominant etiological factor of cirrhosis. Furthermore, 56.5% of patients had a history of regular use of proton pump inhibitors (PPI), possibly because of the increased prevalence of dyspepsia among the cirrhotic due to compromised hepatic parenchymal status, presence of ascites, and gut dysbiosis. The infection patterns among the three groups (i.e., CA, HCA, and NC) are described in Table 1. UTI was the dominant mode of infection among the study population at 22.5% of cases, followed by pneumonia at 20% of cases;14.5% of patients suffered from SBP, and13.5%had skin/soft-tissue infections (SSTIs). The HCA group had a higher frequency of UTI and SBP, whereas the CA group had higher frequencies of pneumonia and SSTI.

**Table 1:** The mode of infection among the three groups in the study.

Mode of infection	Community-acquired (n = 63)		Healthcare-associated (n = 90)		Nosocomial (n=47)		p-value
	n	%	n	%	n	%	
Urinary tract infection	10	15.87	30	33.33	5	10.64	0.034
Spontaneous bacterial peritonitis	9	14.29	15	16.67	5	10.64	0.045
Pneumonia	15	23.81	20	22.22	5	10.64	0.031
Skin/soft-tissue infection	12	19.05	10	11.11	5	10.64	0.078
Spontaneous bacteremia	5	7.94	5	5.56	5	10.64	0.983
Mixed	9	14.29	5	5.56	8	17.02	0.376
Other	3	4.35	5	5.56	14	29.79	0.068

Data on the course of infection among the three groups are described in Table 2. Severe sepsis was more common in the HCA group than in the NC and CA groups. The reason behind this may be that the HCA groups are mostly exposed to the healthcare-related adverse situation, and also might be inadequate precautionary measures adopted by the HCA group.

**Table 2:** The course of infection among the three groups in the study.

	No sepsis (n=51)	Sepsis (n=85)	Severe sepsis (n=64)	p-value
Community-acquired	10 (19.6%)	43 (50.6%)	10 (15.6%)	0.002
Healthcare-associated	30 (58.5%)	27 (31.8%)	33 (51.6%)	
Nosocomial	11 (21.5%)	15 (17.6%)	21 (32.8%)	

The causative organisms of the infections and antibiotic resistance patterns are listed in Table 3. Gram-negative bacilli were the most common causative organisms of infection among the three groups, followed by Gram-positive cocci. Data on different types of antibiotic resistance patterns are given in Table 4, where statistically significant differences(p<0.05) were observed among the three groups.

**Table 3:** Causative organisms and antibiotic resistance patterns among the three groups in our study. NB*: In the “others” category, no growth was observed in the bacterial plates during culturing; therefore, the p-value was not calculated.

Bacterial spectrum analysis	Community-acquired (n=63)		Healthcare-associated (n=90)		Nosocomial (n=47)		p-value
	n	%	n	%	n	%	
Gram-negative bacilli	20	31.7%	30	33.3%	17	36.17%	0.017
Enterobacteriaceae strains resistant to at least one beta-lactam antibiotic	1	1.59	4	4.44	5	10.63%	
Quinolone-resistant strains	2	3.17	4	4.44	7	14.89%	
Gram-positive Cocci	18	28.5 %	15	16.6%	11	23.40%	0.032
Ampicillin-resistantEnterococcus strains	2	3.17	4	4.4%	5	10.63%	
Gram-negative and Gram-positive strains	2	3.17%	1	1.1%	2	4.2 %	0.001
Others*	18	28.57	32	35.55	0	0	

**Table 4:** Types of antibiotic resistance among the three groups in the study. NB*: No resistance to available antibiotics could be observed; therefore, p-values were not compared among the three groups.

Antibiotic spectrum analysis	Community-acquired (n = 63)		Healthcare-associated (n = 90)		Nosocomial (n=47)		p-value
	n	%	n	%	n	%	
Third-generation cephalosporin-resistant strains	26	41.3 %	30	33.3 %	10	21.3 %	0.017
Piperacillin–tazobactam-resistant strains	7	11.1 %	6	6.7 %	5	10.6 %	0.0163
Quinolone-resistant strains	5	7.9 %	5	5.6 %	5	10.6 %	0.024
Non Resistant strains	25	39.68%	49	54.44%	27	57.44	

MELD scores at the time of diagnosis of infection and at the time of hospitalization/admission are given in Table 5. The MELD scores were significantly higher at the time of diagnosis of infection than those at the time of admission among all three groups. Data on the duration of hospitalization among the three groups are given in Table 6, which showed that longer durations of hospitalization were more common in the HA group compared to the CA and HCA groups. Data on infections associated with AKI incidence among the three groups are given in Table 7.

**Table 5:** Comparison of MELD scores among the three groups in the study. *Data are expressed as mean ± SD; #indicates paired t-test results; @indicatesANOVA results.

MELD score	Community-acquired (n = 63)	Healthcare-associated (n = 90)	Nosocomial (n=47)	p-value^@^
At diagnosis of infection	14.2 ± 2.3	15.6 ± 3.9	13.6 ± 3.8	0.057
At Admission	11.2 ± 3.2	12.4 ± 5.9	12.3 ± 7.3	0.071
p- value^#^	0.002	0.001	0.001	

**Table 6:** Duration of hospitalization among the three groups in the study.

Duration of hospitalization	Community-acquired (n = 63)		Healthcare-associated (n = 90)		Nosocomial (n=47)		p-value
	n	%	n	%	n	%	
<10 days	10	15.87	15	16.67	5	10.64	0.081
>10 days	53	84.13	75	83.33	42	89.36	

**Table 7:** Infections associated with acute kidney injury (AKI) among the three groups in the study.

Infection associated A.K.I.	Community-acquired (n = 63)	Healthcare-associated (n = 90)	Nosocomial (n=47)	p-value
A.K.I. stage I	13 (20.6 %)	20 (22.2 %)	8 (17.0%)	0.012
AKI stage II	10 (15.8 %)	10 (11.1 %)	3 (6.3%)	
A.K.I. stage III	7 (11.1 %)	(5.5 %)	4 (8.5 %)	

## Discussion

Cirrhosis patients are more likely to suffer from different infectious agents due to their altered immune system, which can vary among patients, with a perpetual state of constant circulatory dysfunction, an imbalanced coagulation system, and immune deficiency as predisposing factors [12]. Cirrhosis patients have an increased predisposition to various infections due to the overwhelming and aberrant activation of proinflammatory cytokines. Furthermore, these patients exhibit decreased levels of circulating cytokines endotoxins and increased bacterial load due to the development of portosystemic shunts. Thus, the body's systemic inflammatory response is significantly disturbed, contributing to higher infection frequencies. This inappropriate and aberrant rise in infection leads to homeostasis instability and increased morbidity and mortality [13-14].

The mean age of our study participants was 59 years, similar to the study by Bajaj JS et al. 2019 and Ding X et al. 2019 reported studies [7,15], where the mean age at presentation was 57.2 and 55.5 years, respectively. In another study, the mean age of the population was 64 years, which was higher than in the present study [16]. Most of our study participants were men (72.5%), also observed (80.8%, and 68%, respectively) [15,16]. Alcoholic liver disease(ALD) was the most common etiological factor of cirrhosis, followed by non-alcoholic steatohepatitis(NASH), as reported by Bajaj et al. [7]. One study described NASH as the most common etiological factor of cirrhosis, followed by ALD, cryptogenic origins, and Hepatitis C and B viral-related infection [11]. However, chronic viral hepatitis was found to be the most common factor of cirrhosis in our study, as reported elsewhere [15,17]. The mean MELD score was 13.6 ± 3.8 in cases of infection and 15.6 ± 3.9 in cases with infection. One study reported mean MELD scores of 19.05 in cases with no infection, and 22.17 in cases with infection [8], and similar trends were seen elsewhere [18]. In our study, the mean MELD score at baseline was relatively lower than that reported elsewhere (i.e., 20.8±6.7) [11]. 42.5% of our patients had sepsis, which was severe in 32% of patients. Similar findings were observed in other studies in cirrhosis, with one reporting a sepsis prevalence of 47.3%, with 10.75% of patients having severe sepsis or septic shock, and another reporting a sepsis prevalence of 20.67% [11,16].

 In the present study, 36.5% of our patients had AKI associated with NC infection, whereas 47.62% had CA infection. Another study revealed that AKI episodes were more common in cases of NC infection (68%) compared to cases without NC infection (46%) [7]. Furthermore, our results showed that severe sepsis and infection-associated AKI were more common in patients with NC infection than those with CA infection. One explanation for this result is that cirrhosis patients with NC infection might have an excessive cytokine response compared to CA infection cases, as suggested elsewhere [19]. Approximately half of the patients in the current study had a history of long-term PPI usage, which has been associated with an increased risk of bacterial infection in the studies by Barletta JF et al. 2013, Deshpande A et al. 2012 [20-21]. The pathophysiological mechanism involved in long-term PPI usage can be described as an increased chance of intestinal bacterial proliferation and translocation through the leaky gut syndrome and subnormal chemotaxis and bacterial phagocytosis [22]. PPIs have historically been frequently and erroneously overprescribed in cirrhosis [23].

HCA was the most common type of bacterial infection in the present study, followed by CA and NC-a trend that has also been reported in other studies [17,24]. We observed a higher rate of NC infection which is fairly common in cirrhosis, than that reported by Bhattacharya et al. 2019 [11]. However, CA infections were the most common cause of sepsis in cirrhosis in Refs-7 and 15, which contradicts the results of the current study and those mentioned above. The current study found UTI to be the most common (22.5%) type of infection in CA and NC infections, followed by pneumonia (20%), SBP (14.5%), and SSTI (13.5%). These results were similar to those of another study, where UTI was the most common type of infection (53%)in cirrhosis, followed by SBP (44%), pneumonia (28%), and SSTI in 18% [7]. A second study reported that UTI was the most common infection in cirrhosis patients (36.36%), followed by ascitic fluid infections (23.63%)and pneumonia (16.36%) [18]. A third study observed that SBP was the most common infection found in cirrhosis patients (38.65%), followed by UTI (36.97%) and pneumonia (5.88%) [11].

In the current study, Gram-negative organisms belonging to the Enterobacteriaceae family were the most common infective organisms, which was in contrast with results from the study conducted by Bajaj et al. 2019 [7], where Gram-positive organisms were the most common infective organisms in cirrhosis patients. A second study reported that Gram-negative organisms such as E. coli and K. pneumonia were the most common causes of infection in cirrhosis [15]. Usually, CA infections are most commonly caused by Gram-negative organisms, whereas Gram-positive organisms most commonly cause NC infections; currently, Gram-positive cocci infections are on the rise compared to Gram-negative bacteria [25-26]. Third-generation cephalosporin-resistant strains were the most common bacteria encountered in NC and CA infections, followed by quinolone-resistant strains. Some have suggested that third-generation cephalosporins such as cefotaxime are among the most frequently prescribed antibiotics for cirrhosis, which might be responsible for their increased resistance to treat bacterial infection in cirrhosis [17]. Therefore, based on these findings, third-generation cephalosporins should not be ideally used as a first-line therapy in NC and CA infections in cirrhosis. Another study showed that empirical treatment with cefotaxime had a higher incidence of failure in cirrhosis compared to other antibiotics used empirically [27]; therefore, it was suggested that physicians should be much more vigilant and intentional in deciding the optimal empirical treatment for cirrhosis, and the mode of infection (e.g., NC or CA infection) and local epidemiological factors should be considered when choosing of antibiotics

In the current study, 17.5% of participants expired during hospitalization. In addition, cases with NC infection had a higher complication rate and mortality during hospitalization than cases with CA infection. The findings from other studies supported some results of the current study [11,18,28-29]. A mortality rate of 30.2% was found in cirrhosis with sepsis among another study population [15]. Moreover, strain identification and drug sensitivity through molecular dockings, such as carried out for H. pylori [30], are required in cases of NC infection in this region of India for better management of bacterial infection. Therefore, the mortality rate reported in the present study was lower than in previous studies. Routine screening is essential in the early detection of infections, which could lead to significant clinical changes in management strategy and improve the outcome of hospitalized cirrhosis patients.

Limitations of the study

The present study had some limitations. For example, this was a single-center study, and the outcomes might only apply in some places. In addition, this study had a small sample size. Finally, in our study, the most common etiological factor of cirrhosis was an abuse of alcohol; regular alcohol consumption can lead to further immunosuppression in cirrhosis and an increased risk of infection; the above findings may not apply to other etiological factors of cirrhosis.

## Conclusions

As cirrhosis patients are often immunocompromised, and given the erratic and irrational use of PPI, it is difficult to predict and manage the infection patterns among cirrhotic patients. Aiming to address the lack of data on merging bacterial resistance and antibiotic sensitivity patterns both globally and in India, this study attempted to assess the types and designs of bacterial infections, as well as their resistance patterns, in cirrhosis, which could be beneficial in deciding and choosing appropriate antibiotics during patient management, with the ultimate hope of minimizing morbidity and mortality in such cases.
